# Adenosquamous Carcinoma of Skene’s Gland: A Case Report and Literature Review

**DOI:** 10.3389/fonc.2022.893980

**Published:** 2022-08-09

**Authors:** Qian Gao, Xiaoyun Liu, Lin Ye, Tingting Lv, Yanyi Teng, Jian Lan, Tingchao Li, Min Tian, Juqi Chen, Shanshan He, Shengyan Xie, Yan Zou

**Affiliations:** ^1^ Department of Gynecology and Obstetrics, The Third Affiliated Hospital of Zunyi Medical University (The First People's Hospital of Zunyi), Zunyi, Guizhou, China; ^2^ Department of Gynecology, The Third Affiliated Hospital of Zunyi Medical University(The First People's Hospital of Zunyi), Zunyi, Guizhou, China; ^3^ Department of Pathology, The Third Affiliated Hospital of Zunyi Medical University(The First People’s Hospital of Zunyi), Zunyi, China

**Keywords:** skene’s gland carcinoma, skene’s gland adenocarcinoma, paraurethral gland, urethral adenocarcinoma, adenosquamous carcinoma

## Abstract

Skene’s gland is homologous to the male prostate and can secrete prostate fluid. However, Skene’s gland carcinoma is extremely rare, with only 20 cases reported in the literature thus far. We report the first case of adenosquamous carcinoma of Skene’s gland. The patient was a 54-year-old woman who was admitted to our hospital due to vaginal bleeding and a vaginal mass, accompanied by multiple lymph nodes and vulvar metastases. She had a history of dysuria and episodic hematuria for 2 years. Contrast-enhanced pelvic MRI showed a mass in the right labia majora and swelling of the right inguinal lymph nodes. In addition, a mass in the anterior vaginal wall surrounded the urethra and grew in a semi-encircled manner. After receiving neoadjuvant chemotherapy, MRI revealed that the mass surrounding the urethra had shrunk, and the nodular shadow of the right labia majora was larger than before. The patient underwent elective surgery. Surgical pathology showed adenosquamous carcinoma, and immunohistochemistry suggested intestinal differentiation. Forty-six days after surgery, the patient subsequently died of tumor consumption and organ insufficiency due to cerebral infarction, recurrence, and multiple metastases. This paper describes the clinical, radiological, and histopathologic features as well as the prognosis of the rare disease adenosquamous carcinoma of Skene’s gland. In addition, we briefly review the published literature.

## Introduction

Urethral carcinoma is a rare malignant tumor in women, accounting for approximately 0.02% of female cancers and approximately 0.003% of female urogenital malignant tumors, and urethral adenocarcinoma accounts for only 8-10% of the primary female urethral carcinomas (1–3). The origin of urethral adenocarcinomas remains unknown, but female urethral adenocarcinoma is generally considered to originate from Skene’s gland (4–6). Patients with urethral adenocarcinoma have a poor prognosis and a low survival rate (7, 8). Skene’s gland carcinoma is extremely rare, with only a few cases having been reported. When it is difficult to determine whether the tumor originated from Skene’s gland or female urethral carcinoma, immunohistochemical features can be an important clue to identifying the origin. Because Skene’s gland carcinoma may have intestinal differentiation or immunohistochemical characteristics of intestinal differentiation (2, 9).

We present the first case of adenosquamous carcinoma of Skene’s gland. The patient was a 54-year-old woman who presented to our hospital for vaginal bleeding and a vaginal mass, and she had multiple lymph node and vulvar metastases. Forty-six days after the surgery, due to cerebral infarction, recurrence, and multiple metastases, the patient subsequently died of tumor consumption and organ insufficiency.

## Case Report

**Figure 1:**
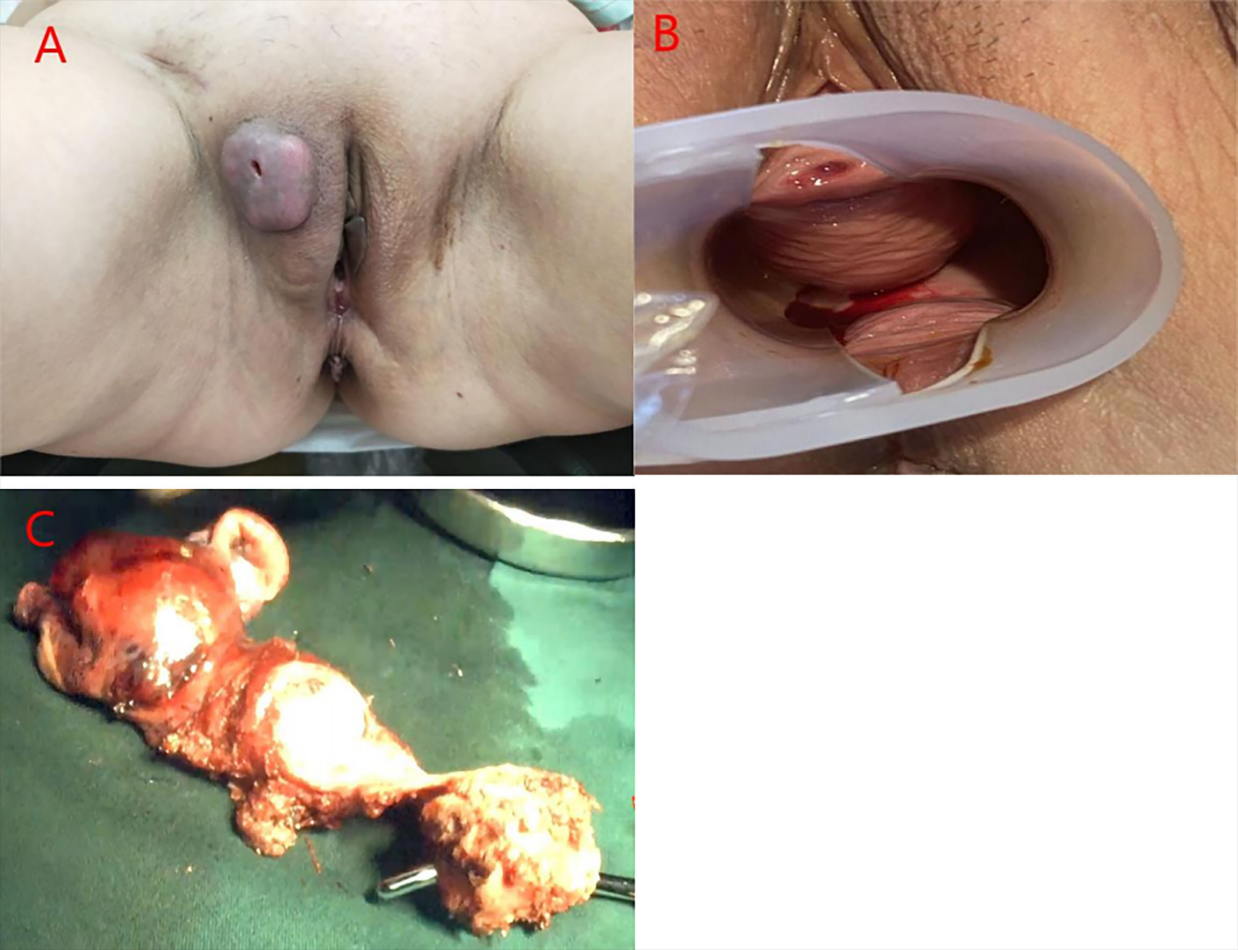
**(A)** A mass of approximately 1.0×1.5 cm was observed in the right labia majora. **(B)** A mass approximately 3.0×3.0 cm was observed in the anterior vaginal wall, and the inner wall of the vagina was smooth. **(C)** Photograph of the gross specimen: the mass in the anterior vaginal wall circumferentially encased the urethra.

**Figure 2:**
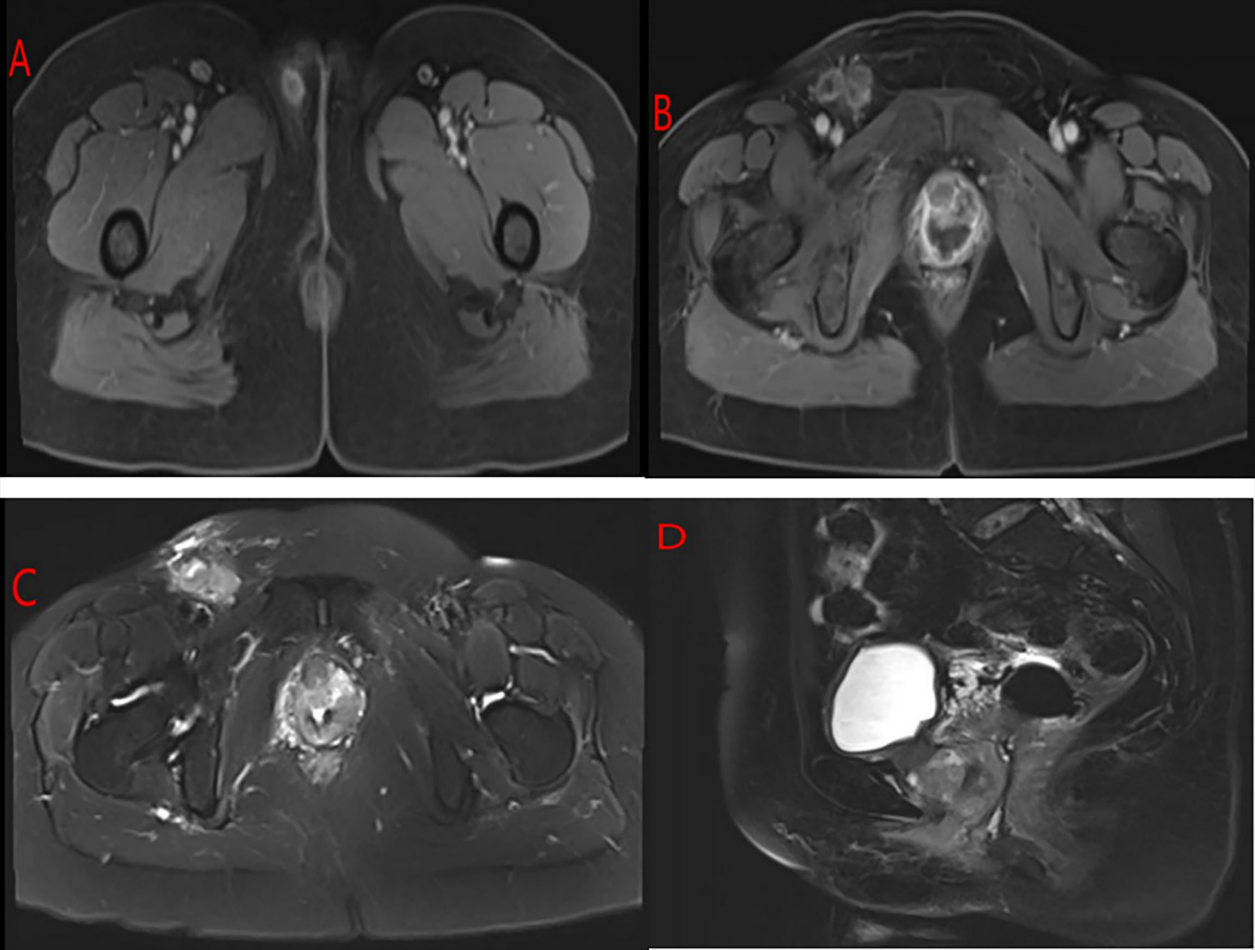
MRI of the pelvis of the patient. **(A)** A mass in the right labia majora. **(B, C)** The right inguinal area showed swelling, multiple lymphadenopathies, and a mass in the anterior vaginal wall that surrounded the urethra and grew in a semi-encircled manner. **(D)** The mass in the anterior wall of the vagina was suspected to invade the urethra but only. Surrounded the urethra.

A 54-year-old woman was admitted to our hospital with irregular vaginal bleeding for 6 months and a protruding vaginal mass detected on one day. She had been suffering from dysuria and episodic hematuria for the past 2 years. The patient had no previous history of gastrointestinal disease or malignancy, and her family history was normal. Physical examination revealed a mass of approximately 1.0×1.5 cm in the right labia majora (**Figure 1A**), a mass of approximately 3.0×3.0 cm in the anterior vaginal wall (**Figure 1B**), and enlarged right inguinal lymph nodes. B-scan ultrasonography showed two hypoechoic nodules in the right groin and a cystic-solid nodule on the right labia majora of approximately 1.4x0.9 cm. In addition, abdominal ultrasonography showed that the liver, gall bladder, pancreas, spleen, and kidney were normal. A chest CT scan showed no abnormalities. Contrast-enhanced CT of the abdomen also revealed no abnormalities. Contrast-enhanced pelvic MRI showed a mass in the right labia majora (**Figure 2A**) and swelling of the right inguinal lymph nodes (**Figures 2B, C**). In addition, a mass was discovered in the anterior vaginal wall that surrounded the urethra and grew in a semi-encircled manner (**Figures 2B–D**). The result of pelvic CT was the same as contrast-enhanced pelvic MRI. CA199 was 250.7 U/ml, whereas other tumor markers were within the normal limits. Biopsy of a right inguinal lymph node demonstrated metastatic squamous carcinoma. Before treatment, we had many discussions with multidisciplinary team (MDT). Our multidisciplinary team including a gynecologist, a urologist, a pathologist, a radiologist, and an oncologist was created. Our multidisciplinary team considered the possibility of malignancy, and the vaginal mass was closely related to the urethra. Thus, we employed the TC regimen (paclitaxel in combination with carboplatin) as neoadjuvant chemotherapy. Three cycles of neoadjuvant chemotherapy were administered, and the pelvic MRI re-examination revealed that the mass surrounding the urethra had shrunk and that the nodular shadow of the right labia majora was larger than before.

**Figure 3:**
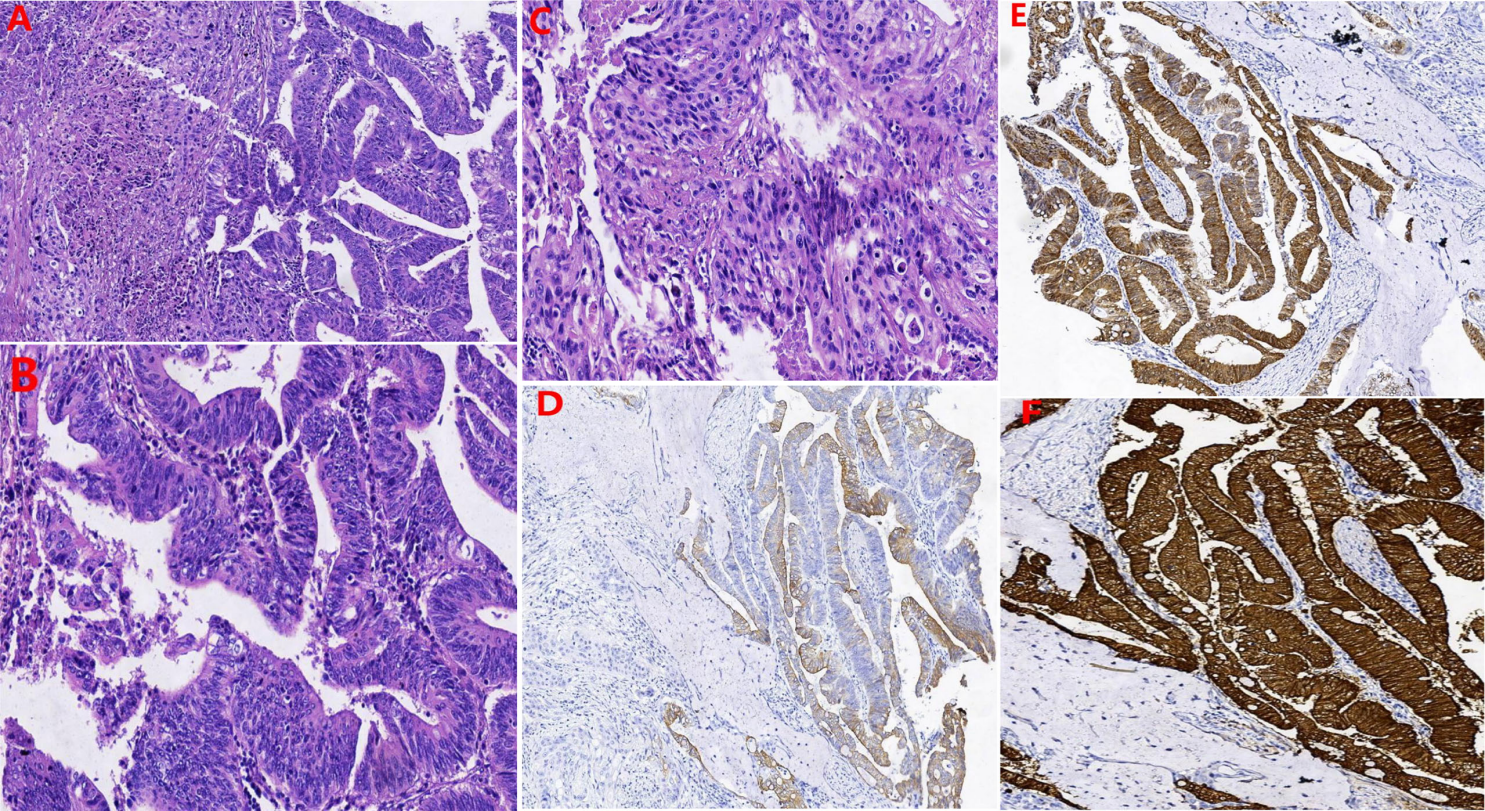
The results of hematoxylin-eosin staining: **(A)** Both glandular and squamous components were present. **(B)** the glands in the adenocarcinoma area were cribriform, papillary arrangements and had patterns of invasive growth. **(C)** The area of squamous carcinoma displayed nest distribution, patchy necrosis, pathological nuclear division, and invasive growth. Immunohistochemically: the tumor cells were positive for **(D)** CK20, **(E)** CDX-2, and **(F)** Villin.

Our multidisciplinary team decided to perform surgery, which included total hysterectomy and bilateral adnexectomy, pelvic lymphadenectomy, lymphadenectomy of the presacral area, para-aortic lymphadenectomy (level A), urethrectomy, total vaginectomy, cystostomy, and partial vulvectomy. After the operation, the specimens were immediately dissected and visually assessed. No lesions were seen in the uterus. The mass in the anterior vaginal wall circumferentially encased the urethra (**Figure 1C**) and was confirmed as adenosquamous carcinoma (**Figures 3A–C**) by histopathological examination. The periurethral tumor invaded only the submucosa of the urethra without involvement of the vaginal wall and was confined to Skene’s gland. Pathology results confirmed that the mass in the right labia majora was squamous carcinoma, and the patient had lymph node metastases (There were 18 pelvic lymph nodes, 3 of which showed metastasis (3/18). There were 20 para-aortic lymph nodes, 1 of which showed tumor metastasis (1/20), and 4 presacral lymph nodes without tumor metastasis (0/4).). Immunohistochemically, the tumor cells were positive for CK20 (**Figure 3D**), CDX-2 (**Figure 3E**), villin (**Figure 3F**), CK5/6, CK7, and p40 and negative for both PSA and PSAP.

**Figure 4:**
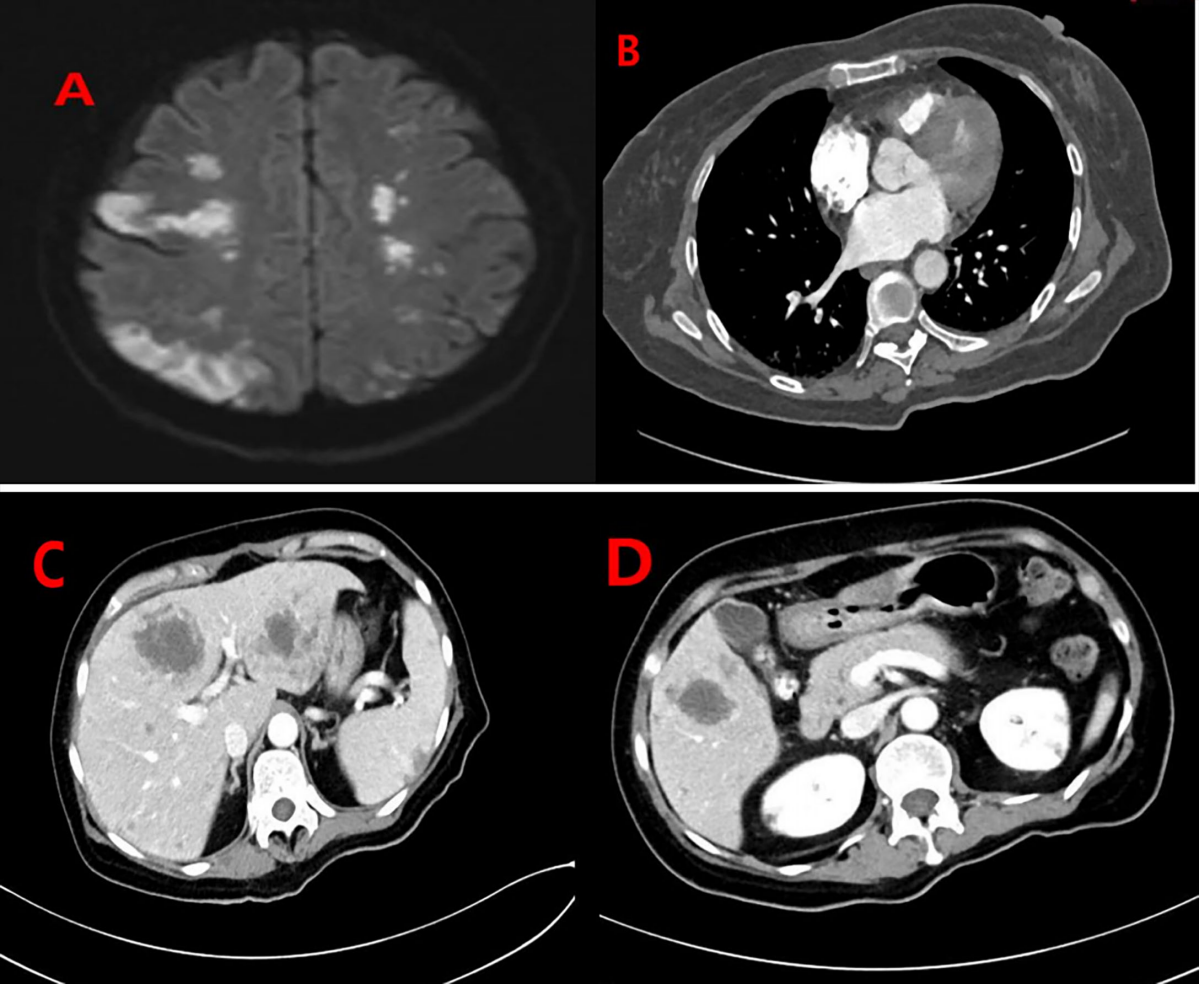
**(A)** MRI of the brain showed multiple lesions in the bilateral brain representing cerebral infarctions. Contrast-enhanced whole-body CT of the patient: **(B)** Filling defects in the segmental pulmonary artery of the right lower lobe were also seen, indicating the presence of thromboemboli. **(C, D)** There were multiple lesions in the abdominal cavity (involving the kidneys, liver, spleen, left adrenal gland, and left pelvic side and bilateral inguinal lymph nodes).

Thirty-two days after the operation, the patient had sudden impaired consciousness, facial paresis, aphasia, and other symptoms. Cerebral embolism was considered. MRI of the brain (**Figure 4A**) showed multiple cerebral infarctions, and contrast-enhanced whole-body CT (**Figures 4B–D**) revealed pulmonary embolism and multiple metastases in the abdominal cavity (involving the kidneys, liver, spleen, left adrenal gland, and left pelvic side and bilateral inguinal lymph nodes). The patient was immediately transferred to the ICU and given hepatoprotective, anticoagulation, and other symptomatic therapy. Subsequently, the patient’s family refused ICU treatment and was transferred back to the gynecology unit. From that point, the patient’s condition deteriorated. The patient died of tumor consumption and organ insufficiency at day 46 following surgery.

## Discussion

The concept of “the female prostate” was first proposed by De Graaf in 1672 (10). In 1880, Alexander J. C. Skene described Skene’s gland and reported that there were two ducts tightly related to the glands in the female periurethral area; since then, the paraurethral gland has been called Skene’s gland (11). Skene’s gland is homologous to the male prostate and can secrete prostate fluid (2). However, some cases reported to be Skene’s gland carcinoma have been positive for PSA, and some have been negative for PSA. Therefore, PSA lacks diagnostic specificity in Skene’s gland carcinoma (12). Adenosquamous carcinoma of the prostate was first described by Thompson in 1942 (13) and is defined as containing both glandular and squamous histological components (14). However, no previous cases of adenosquamous carcinoma of Skene’s gland have been reported.

The origin of urethral adenocarcinomas remains unknown, but female urethral adenocarcinoma is generally considered to originate from Skene’s gland (4–6). Skene’s gland carcinoma is extremely rare, with only 20 cases reported in the literature thus far (**Table 1**) (1, 2, 5, 6, 9, 12, 15–25). The average age was 70.35 years old with a range between forty-six and eighty-eight years old. The most common presenting symptom was periurethral or urethral lesion (7 cases) followed by hematuria (5 cases), dysuria (3 cases), and urinary incontinence (2 cases). Our patient was admitted to our department mainly due to irregular vaginal bleeding for 6 months. Moreover, she had been suffering from dysuria and episodic hematuria for the past 2 years.

**Table 1:** Summary of cases of Skene's gland carcinoma reported to date.

References	Age	First Sympto	Metastases	Pathology	IHC+marker	Treatment	Outcomes
Klotz (15)	67	Dysuria	No	Mucous adenocarcinoma	NA	Anterior pelvic exenteration	Convalescence and good follow-up at 6 months
Zaviacic (16)	70	No	Lungs, liver, lymph nodes and the rib	Adenocarcinoma with cribriform multiple gland-like lumen	PSA+, PSAP+	No	Autopsy examination, died of cardiorespiratory failure while hospitalized
Dodson (5)	70	No	No	Adenocarcinomawith a distinct cribriform pattern	PSA+, PSAP+	Wide excision and bilateral inguinofemoral lymphadenectomy	NA
Ali (17)	50	Vaginal bleeding and pai	No	Infiltrating adenoid cystic carcinoma with prominent perineural invasion	NA	a radical excision of the mass with partial vulvectomy and right groin node dissection with external beam radiation	No evidence of disease at 26 months follow-up
Sloboda (6)	46	Stress incontinence and urethritis	Left inguinal lymph nodes	Adenocarcinoma with cribriform multiple gland-like lumens	PSA+, PSAP+	Local excision	NA
Tregnago (2)	87	A bleeding urethral polyp	No	Adenocarcinoma resembling prostate, Gleason score 4+4=8	PSA+,P501S+ NKX3.1+ AMACR+	Local excision	No recurrence after 8 months of follow-up
Tregnago (2)	61	Urethral polypoid lesion	No	Adenocarcinoma resembling prostate, Gleason score 4+4=8	PSA+,P501S+ NKX3.1+,CK20+	Local excision	Asymptomatic after 4 months of follow-up
Kyriazis (12)	71	A urethral mass	No	Adenocarcinoma, composed of fused and coalescent gland	PSA+,CK20+	Local excision	alive without any signs of disease recurrence or metastatic disease at 31 months
Kalinderi (1)	77	Macroscopic hematuria	No	Adenocarcinoma	Cytokeratin AE1/AE3+,PAX8+ AMACR+ CD31+	An anterior exenteration	NA
Lenz (18)	73	Recurrent urinary tract infection	No	Adenocarcinoma consisting of fused irregular cribriform glands	PSA+,PSAP+ NKX3.1+ AMACR+,cytoker -atinAE1/AE3+, cytokeratin 18+	Transurethral resection and patient refused additional treatment and follow u	NA
Kunc (19)	78	Hematuria, dysuria	NA	Adenocarcinoma resembling prostate, Gleason score 4+3=7	PSA+,Prostein+ AR+,CAM 5.2+	NA	NA
Kaufman (20)	69	Vaginal pain, dysuria and nocturia	Pelvic lymph nodes	Adenocarcinoma resembling prostate, Gleason score 4+5=9	PSA+, PSAP+,AR+,CAM 5.2+,NKX3.1+	androgen deprivation therapy (ADT)	NA
Slopnick (21)	76	An enlarging left inguinal mass	Left inguinal lymph node, left obturator lymph node	Adenocarcinoma resembling prostate, Gleason score 4+4=8	CAM5.2+, NKX3.1,pan-kerati n AE1/AE1+,	androgen deprivation therapy (ADT)	the patient was well at 24 months follow-up

IHC indicates immunohistochemistry; NA, not available.

The literature regarding its metastasis and recurrence is limited. Of the 20 cases previously reported, only 4 had metastasis (1-left inguinal lymph nodes metastases; 1-pelvic lymph nodes; 1-left inguinal lymph node metastases and left obturator lymph node; 1-multiple metastasizes including lungs, liver, lymph nodes, and the rib.) However, female urethral adenocarcinoma usually spreads to the perineal skin, vulva, bladder, and vagina and can lead to distant metastasis involving organs such as the lung, liver, bone, and brain in advanced stages (26). Urethral adenocarcinoma arising from Skene’s gland is more common in the distal urethra (12, 17). Adenocarcinoma of the distal urethra easily metastasizes to inguinal lymph nodes proximal to pelvic lymph nodes (27). Urethral adenocarcinoma, which is prone to metastasis (28), has a high recurrence rate (8, 29). Our case had metastasis to the vulvar and inguinal lymph nodes, pelvic lymph nodes, and para-aortic lymph nodes. CT 1 month after surgery revealed multiple metastases in the abdominal cavity (involving the kidneys, liver, spleen, left adrenal gland, and left pelvic side and bilateral inguinal lymph nodes). Thus, adenosquamous carcinoma of Skene’s gland may be prone to metastasis and relapse.

This rare entity needs to be diagnosed with histopathology, and MRI and cystoscopy serve as the auxiliary tools for its diagnosis (12, 23, 24). Korytko et al. (23) reported that Skene’s gland carcinoma may show a characteristic image on MRI: it circumferentially encases the urethra as if it were the male prostate. The unique MRI appearance is helpful for the imaging-based diagnosis of Skene’s gland carcinoma. In this case, pelvic MRI showed a mass in the anterior vaginal wall that surrounded the urethra and grew in a semi-encircled manner, which was consistent with the literature.

Microscopically, the tumor cells of Skene’s gland adenocarcinoma are consistent with prostatic acinar adenocarcinoma (2, 21, 22). In our case, the glands in the adenocarcinoma area were cribriform and papillary and had patterns of invasive growth, and the architecture of the tumor cells was analogous to a Gleason score 4 + 4 = 8 prostatic adenocarcinoma. The area of squamous carcinoma displayed nest distribution and invasive growth.

Skene’s gland carcinoma may have intestinal differentiation or immunohistochemical characteristics of intestinal differentiation (2, 9). There is no literature to analyze and compare the difference between urethral carcinoma and Skene’s gland carcinoma. However, in 2017, Mariko Muto (9) proposed that although the most definite method to determine the origin of the tumor is to find *in situ* lesions, it is often difficult to find *in situ* lesions. When it is difficult to determine whether the tumor originated from Skene’s gland or female urethral carcinoma, immunohistochemical features can be an important clue to identifying the origin. In our case, the tumor cells were positive for CK20, CDX-2, and villin, which suggested intestinal differentiation. Furthermore, the pathology results confirmed that the periurethral tumor only invaded the submucosa of the urethra without involvement of the vaginal wall and was confined to Skene’s gland, and there was no evidence of primary gynecological or gastrointestinal malignancy. These findings support the diagnosis of primary adenosquamous carcinoma of Skene’s gland.

In recent years, the molecular genetics of the Skene’s gland adenocarcinoma has received increased attention. At present, three studies have examined the genomics of the Skene’s gland adenocarcinoma. In 2020, Lenz et al. (18) detected a mutation and loss of heterozygosity of the phosphatase and tensin homolog (PTEN) gene using Illumina Trusight Tumor 170 next-generation sequencing technology, and the molecular genetic characteristics of Skene’s gland adenocarcinoma were described for the first time. The cases reported by Saeed (22) showed loss of PTEN expression but no ERG overexpression. No loss of PTEN expression was detected in Slopnick’s study (21). PTEN and ERG mutations are the two of the most common mutations in prostate adenocarcinoma (22). The PTEN mutation of the Skene’s gland adenocarcinoma strengthens the link with prostate cancer and the status of PTEN may serve as a predictive biomarker and help guide individualized treatment in the future (21, 22).

The incidence of Skene’s gland carcinoma is extremely low, and there is no consensus on the treatment. Local or expanded surgical excision, radiotherapy, and adjuvant androgen deprivation therapy (ADT) were previously used (18, 21, 22). And there was no recurrence after treatment. Only one case was confirmed by autopsy examination after death, and there were no other secondary deaths were reported (16, 21). Given the inherent levels of castrated testosterone in normal postmenopausal women, Skene’s gland carcinoma may be treated as castration-resistant prostate cancer (21). However, our patient was accompanied by multiple lymph node and vulva metastases, so it is considered to be treated as metastatic castration-resistant prostate cancer (mCRPC). Docetaxel can benefit the survival time and quality of life of patients with metastatic castration-resistant prostate cancer. Docetaxel has become the standard chemotherapeutic agent for treating metastatic castration-resistant prostate cancer (30, 31). However, patients may be forced to discontinue docetaxel due to disease progression, unacceptable toxicity, or adverse reactions (32, 33). Early clinical trials demonstrated showed that platinum analogues have modest antitumor activity in patients with advanced prostate cancer. In the clinical study of platinum analogues in combination with other chemotherapeutic agents, the synergistic effect of carboplatin and paclitaxel was determined based on the activity of microtubule-targeting agents in prostate cancer (31). Some scholars have proposed that the combination of paclitaxel and carboplatin has significant efficacy and acceptable toxicity in the study of castration-resistant prostate cancer (31, 33, 34). Paclitaxel in combination with carboplatin (TC regimen) is the commonly used chemotherapy regimen for gynecologic malignancies (35). The combination of carboplatin and paclitaxel is considered is widely regarded as the standard first-line treatment for epithelial ovarian cancer (36), recurrent or metastatic endometrial cancer (37), and non-small cell lung cancer (38). Furthermore, the combination of carboplatin and paclitaxel may represent a very active and potentially less toxic regimen to be used in neoadjuvant chemotherapy. Thus, we employed the TC regimen as neoadjuvant chemotherapy. Three cycles of neoadjuvant chemotherapy were administered, and the pelvic MRI re-examination revealed that the mass surrounding the urethra had shrunk (from 3.3x3.5cm to 3.2x1.5cm). And the chemotherapy regimen was considered effective. A study published in Lancet demonstrated that early palliative care for patients with advanced cancer can improve not only quality of life but also satisfaction in the late stage (39). Therefore, our patient received palliative treatment, and neoadjuvant chemotherapy was first given to reduce tumor load. Considering the rarity of this case and the fact that complete tumor resection was essential to determine prognosis, we have performed palliative surgery for the patient after chemotherapy. Nevertheless, 46 days after surgery, the patient died due to disease recurrence and metastasis. Adenosquamous carcinoma of the prostate is considered to be a variant of aggressive prostate cancer, which has an unfavorable prognosis and a low survival rate, and the majority of these patients die within a year after diagnosis (14, 40, 41). However, compared with the previously reported Skene’s gland adenocarcinoma cases, our case had a worse prognosis, suggesting to some extent that adenosquamous carcinoma of Skene’s gland is more aggressive than Skene’s gland adenocarcinoma and may be a variant of aggressive Skene’s gland carcinoma.

## Conclusions

In conclusion, we report the first case of adenosquamous carcinoma of Skene’s gland. In our case, the architecture of the tumor cells is analogous to a Gleason score 4 + 4 = 8 prostatic adenocarcinoma. In the previously reported Skene’s gland carcinoma, few patients were accompanied by metastasis, and there was no recurrence after treatment. Only one case was confirmed by autopsy examination after death, and there were no other secondary deaths were reported. Despite appropriate surgery combined with neoadjuvant chemotherapy, the patient still relapsed, experienced rapid metastasis, and had a poor outcome. Accordingly, we propose that adenosquamous carcinoma of Skene’s gland may have higher malignant potential than Skene’s gland adenocarcinoma and may be a variant of aggressive Skene’s gland carcinoma.

More reports and data in the future will aid further understanding of adenosquamous carcinoma of Skene’s gland.

## Data Availability Statement

The original contributions presented in the study are included in the article/supplementary material. Further inquiries can be directed to the corresponding author.

## Ethics Statement

Written informed consent was obtained from the individual(s) for the publication of any potentially identifiable images or data included in this article.

## Author Contributions

QG drafted the manuscript. QG, LY, and TLv collected clinical data. SH, SX, and YZ took care of the patient. QG, TLi, MT, and JC provided and analyzed the pathological information. QG, YT, JL and XL designed the study and revised the manuscript. All authors contributed to the article and approved the submitted version.

## Conflict of Interest

The authors declare that the research was conducted in the absence of any commercial or financial relationships that could be construed as a potential conflict of interest.

## Publisher’s Note

All claims expressed in this article are solely those of the authors and do not necessarily represent those of their affiliated organizations, or those of the publisher, the editors and the reviewers. Any product that may be evaluated in this article, or claim that may be made by its manufacturer, is not guaranteed or endorsed by the publisher.
